# The Double Jeopardy of Feeling Lonely and Unimportant: State and Trait Loneliness and Feelings and Fears of Not Mattering

**DOI:** 10.3389/fpsyg.2020.563420

**Published:** 2020-12-17

**Authors:** Sarah E. McComb, Joel O. Goldberg, Gordon L. Flett, Alison L. Rose

**Affiliations:** Department of Psychology, York University, Toronto, ON, Canada

**Keywords:** loneliness, mattering, anti-mattering, friendships and peer relationships, peer isolation

## Abstract

There have been recent concerns about an “epidemic of loneliness” during the pandemic, given the pervasiveness of loneliness in the population and its harmful effects on health and well-being. Therefore, it is important to establish the correlates of loneliness. The purpose of the current study was to explore how loneliness relates to a construct termed mattering, which is the feeling of being important to other people. Mattering was assessed with multiple measures in the current study (e.g., mattering in general, fears of not mattering, and mattering to peers). A sample of 172 female psychology undergraduate students aged 18–25 years completed self-report measures of general mattering, mattering to peers, anti-mattering, fear of not mattering, and state and trait loneliness. As predicted, lower levels of both general mattering and mattering to peers were associated with higher state loneliness. Higher feelings of anti-mattering (feelings of being invisible and insignificant to others) and fears of not mattering were associated with greater trait loneliness, as well as a reduced sense of mattering to friends. The findings illustrate that feeling as though one does not matter to others (i.e., feeling insignificant and unimportant) is associated with increased state and trait loneliness among young women. Implications are discussed for loneliness theory and how these results can enhance both clinical understanding and practice.

## Introduction

Loneliness is the distressing feeling that arises when one’s desired quantity or quality of social connection has failed to be met (Peplau and Perlman, 1982). In recent years, concerns have been expressed by public health officials and researchers about an “epidemic of loneliness” given the pervasiveness of loneliness across the population and its debilitating effects (Holt-Lunstad, 2017; King, 2018). A recent study conducted in Canada found that 48% of Canadian adults reported feeling lonely, and 62% said they wished their friends and family would spend more time with them (Angus Reid Institute, 2019). Feelings of loneliness are present across the lifespan from adolescence through to old age, and while loneliness is often thought to be a condition that primarily afflicts the elderly, research polling has actually found that young women under the age of 25 report being the loneliest demographic group (Angus Reid Institute, 2019) and are thought to show particular susceptibility (Rokach, 2000). Despite young women being at high risk for experiencing loneliness very little research has been specifically conducted on experiences of loneliness and correlates of loneliness among young women, which is a gap the current study intends to address. Research on correlates of loneliness represents an important area of research given the negative consequences of loneliness, such as increased risk of depression, anxiety, poor immune functioning, and physical health, as well as earlier mortality (Uchino, 2006; Lasgaard et al., 2011; Cacioppo and Cacioppo, 2014; Hostinar et al., 2014; Rokach, 2019). Indeed, a recent study of patients receiving community mental health services found that loneliness predicted subsequent levels of four mental health indicators and was a better predictor than objective social isolation and social capital (see Wang et al., 2020).

Previous research has established that several interpersonal variables are associated risk factors for trait loneliness. Lack of social connection, such as living alone, being unmarried, not participating in social groups, and having fewer friends to turn to in times of need have all been associated with greater feelings of loneliness (Holt-Lunstad et al., 2010). Additionally, lack of social support from family, and especially from friends, was correlated with greater feelings of loneliness among young adults (Lee and Goldstein, 2016; Chang et al., 2017). Further, poor attachments to mothers and fathers, and especially peers, were all related to greater loneliness, as was low feelings of general belongingness (Yildiz, 2016).

Taken together, past research seems to reflect that feelings of belonging and social information from others regarding levels of interpersonal support, connection, and one’s relative worth have an influence on trait loneliness. It stands to reason that feelings of mattering to others then may also be a relevant interpersonal predictor of loneliness. Mattering can be defined as the sense that other people find us important, depend on us, are interested in us, and care about what happens to us (Rosenberg and McCullough, 1981). If a person feels unimportant to others and as though others are not interested in them or care about what happens to them (i.e., he/she feels as though he/she does not matter to others), it follows that they may then feel a sense of loneliness.

Below we discuss mattering and loneliness from a historical perspective and a contemporary perspective. First, however, we describe the mattering construct in more detail. Rosenberg and McCullough (1981) introduced a form of relational mattering through their focus on the feeling and the need to be important to other people. The person who feels like she or he matters is someone who feels seen and heard by others who are valuing them. It is related to but more specific than Leary’s notion of the sociometer, which blends being valued with others with openly being rejected or accepted by others and how this can impact self-esteem (see Leary, 2012; Leary and Acosta, 2018).

Key nuances help distinguish mattering from other constructs. For instance, Prilleltensky (2020) introduced an expanded view that sees mattering as being someone who is both valued by other people and who gives value to other people. This last component is important because it makes mattering less reactive and helps distinguish it from other constructs because people can become “mattering agents” who promote feelings of mattering among people in their social circle (for a related discussion, see Flett, 2018). Most notably, mattering is distinguishable from related constructs. It has been shown empirically in several studies that mattering is related to but distinct from self-esteem (e.g., Rosenberg and McCullough, 1981; Elliott et al., 2004; Dixon and Robinson Karpius, 2008; Flett et al., 2016b). A recent investigation by Flett and Nepon (2020) found that mattering and self-esteem were associated positively but mattering predicted significant variance in depression beyond self-esteem. Indeed, in their original paper, Rosenberg and McCullough (1981) showed in four samples of adolescents that after controlling for self-esteem, mattering was still a predictor of reduced levels of depression. There is also extensive evidence to support the conceptual and empirical distinction between mattering and related concepts such as belongingness and social support (see Elliott et al., 2004; Elliott, 2009; Flett, 2018). The distinction between the need to matter vs. the need to belong is perhaps best reflected by the person who feels part of a broader group and thus belongs but is not valued or extensively noticed within the group and thus feels a sense of not mattering to others.

### Mattering and Loneliness

At present, to our knowledge, only one study thus far has examined the association between mattering and loneliness. Flett et al. (2016a) examined loneliness and mattering as part of a broader study. A sample of 232 undergraduate students completed various measures that included a measure of trait loneliness and the five-item General Mattering Scale (Marcus and Rosenberg, 1987). A robust negative association was found between mattering and loneliness (*r* = −0.65), thus confirming that low feelings of general mattering to others was associated with greater trait loneliness among both men and women. Additional results indicated that feelings of not mattering partially mediated the link between a reported history of maltreatment and loneliness.

The current article re-examines and extends this association between feelings of not mattering and loneliness with multiple measures of each construct. While there has not been an extensive theoretical emphasis thus far on loneliness and mattering seen through a conceptual lens, some useful insights have been provided by Erich Fromm (1941). Flett (2018) describes in his broad review on the psychology of mattering, the influence that Fromm had on the mattering field and how his insights deserve more emphasis not only due to their influence on Rosenberg and McCullough (1981). Most notably, for our purposes, Fromm (1941) proposed that feelings of powerlessness (written presciently in the context of world events at the time) and personal insignificance (i.e., feelings of not mattering) are closely intertwined with feelings of loneliness. Indeed, the mandated exposure of individuals to social isolation around the globe due to the corona virus-related public health crisis has reignited the need to explore correlates of loneliness and populations at specific risk (Flett and Zangeneh, 2020). Specifically, Flett and Zangeneh (2020) describe the vulnerability to psychological pain of those people who are alone and who feel unimportant, that their lives lack significance. Given these historical and contemporary observations, there is a clear need for programmatic research on loneliness and feelings of not mattering.

The current research was based on an expanded view of loneliness (i.e., state and trait loneliness) and mattering. We went beyond mattering in general to also assess mattering with respect to a specific relationship. To our knowledge, this topic has not been examined in terms of how feelings of mattering to specific others (e.g., mother, father, and peers) is related to loneliness. Given past research that has documented stronger associations between loneliness and lack of peer support or attachment, it is anticipated that low feelings of mattering to friends may be an especially important predictor of loneliness among young women and go beyond studies of basic belongingness in this population (Asher and Weeks, 2014).

We also examined mattering with additional focus on two recent extensions of the construct. The concept of anti-mattering is described in Flett (2018) and is evaluated using items that emphasize not mattering and feeling marginalized. According to Flett (2018), feelings of anti-mattering, are distinct from, and not merely the opposite of mattering, in keeping with the notion that constructs such as optimism and pessimism and hope vs. hopelessness are not mere endpoints of the same continuum. A recent review has summarized extensive evidence attesting to the incremental validity of this new measure of anti-mattering when considered along with the General Mattering Scale (see Flett and Nepon, 2020). Accordingly, we include a new measure of anti-mattering to evaluate whether it is also a relevant interpersonal correlate of loneliness. Anti-mattering can be defined as feeling insignificant and invisible to others and feeling as though no one cares about what you have to say or think. Those who strongly endorse feelings of anti-mattering may feel as though they do not matter at all to anyone. It stands to reason that those who feel as though they do not matter to anyone would be more likely to be dissatisfied with their social relationships and to feel lonely.

Further, we included a new measure of fear of not mattering to others that reflects the anxiety that people have about the possibility that they will not matter to others. This concept has been described by Casale and Flett (2020) as distinguishable from other interpersonally-based fears such as a fear of missing out or separation fears. The fear of not mattering is similar to other interpersonally-based constructs (e.g., rejection sensitivity, reassurance seeking, and fear of negative evaluation) in that it reflects unmet interpersonal needs and a need for validation through connection from others, but it is a specific fear reflecting a concern about not being valued and not being seen or heard by other people who show little interest. This emphasis on a fear of being or becoming insignificant to others is in keeping with research that ties individual differences in feelings of not mattering to others with anxious forms of insecure attachment. More generally, the fear of not mattering reflects the overlap that exists between the mattering and anxiety constructs and the anxious arousal and evaluation apprehension that accompanies a sense of not mattering (see Flett, 2019). Fear of not mattering to others may reflect a ruminative preoccupation about the threat of the depreciation of one’s worth or value to others and the loss of important social relationships or resources. This fear of not mattering might be experienced by people who have lost people in their lives who were key sources of feelings of mattering. This type of fear could be relevant for older adults given the suggestion from Rosenberg and McCullough (1981) that feelings of mattering may be particularly relevant in understanding elderly people. Given these observations, fear of not mattering to others may be another correlate of loneliness. Certainly past research has found that rumination is associated with greater loneliness among young adults (Gan et al., 2015; Borawski, 2019).

### The Current Study

In summary, the current study aimed to expand the sparse literature on how mattering is related to feelings of state and trait loneliness using a sample of young women aged 18–25 years old since they are considered to be the loneliest demographic group. The purpose of the current study was to examine how feelings of general mattering, mattering to peers, anti-mattering, and fear of not mattering related to reports of trait and state loneliness. Based on past research, the following hypotheses were tested: (1) Feelings of both general mattering and mattering to peers will be negatively associated with state and trait loneliness and (2) Feelings of anti-mattering and fear of not mattering will both be positively associated with state and trait loneliness. These hypotheses were based on past research. Our emphasis on going beyond trait loneliness to also consider state loneliness reflected our interest in assessing very current feelings of loneliness that could perhaps more closely reflect the current experience of young women as they adapt to the pandemic and its various challenges. It also reflects past research attesting to the usefulness of examining state loneliness as a form of reactivity in specific contexts (see van Roekel et al., 2018).

## Materials and Methods

### Participants

Participants were 172 female psychology undergraduate students recruited through an online research participant pool at York University in Toronto, Canada. Inclusion criteria included being a biological female and being between the ages of 18–25 years. As previously mentioned, this sample was chosen because women in this age bracket have reported being the loneliest demographic group (Rokach, 2000; Angus Reid Institute, 2019). Participant ages ranged from 18–25 years (*M* = 19.20, *SD* = 1.68). The self-reported ethnic distribution of the sample was 34.7% Caucasian, 28.9% South-Asian, 10.4% East Asian, 9.8% Middle Eastern, 5.8% Black/African-Canadian, 1.2% Pacific Islander, 0.6% Hispanic/Latino, and 8.1% identified as “Other.” Most participants (71.1%) reported completing a high school diploma as their highest level of completed education, followed by 19.7% who completed some college, 4.0% who completed a 2-year degree, 4.0% who completed a 4-year degree, and 0.6% who had completed a doctorate.

### Measures

#### The General Mattering Scale

The General Mattering Scale (GMS; Marcus and Rosenberg, 1987) is a brief five-item self-report scale that is used to measure how much one perceives that they matter to others at an overall level. Participants are asked to indicate how much they agree with each statement, such as “How important do you feel you are to other people?,” by responding on a scale from 1 = *not at all* to 4 = *a lot*. Higher scores indicate greater perceived mattering. Internal consistency in the current study was good (*α* = 0.78). Extensive evidence attests to the reliability and validity of this measure (see Flett, 2018).

#### The Mattering to Others Questionnaire

The Mattering to Others Questionnaire (Marshall, 2001) is an 11-item self-report scale that can be used to assess perceived mattering to one’s mother, father, or friends. We focused solely on assessing levels of mattering to friends/peers in the current study. Participants are asked to indicate how much they agree with each statement, such as “I matter to my friends,” by responding on a scale from 1 = *not much* to 5 = *a lot*. A mean score for all items are calculated and higher scores indicate greater perceived mattering. The level of internal consistency in the current study was somewhat low (*α* = 0.59), which is uncharacteristic of the measure given that there is extensive psychometric support for this scale (see Flett, 2018).

#### The Anti-Mattering Scale

The Anti-Mattering Scale (AMS; Flett, 2018) is another brief five-item self-report scale that is used to measure anti-mattering at an overall level. The measure is designed to parallel the format of the GMS. Anti-mattering can be defined as a feeling of being insignificant and invisible to others. Participants are asked to “*please choose the rating that you feel is the best for you*,” in regards to each item (e.g. “*How much do you feel like you do not matter?*”), by responding on a scale from 1 = *not at all* to 4 = *a lot*. Higher scores indicate greater perceived anti-mattering. Internal consistency in the current study was surprisingly low (*α* = 0.58).

#### The Fear of Not Mattering Inventory

The Fear of Not Mattering Inventory (Besser et al., 2020) is a brief five-item self-report scale that is used to measure fear about not mattering to others. Participants are asked to indicate how much they agree with each statement, such as “To what extent are you afraid that you will not matter to other people?,” by responding on a scale from 0 = *not at all* to 3 = *almost all of the time*. Higher scores indicate greater fear of not mattering. Internal consistency in the current study was excellent (*α* = 0.91).

#### UCLA Loneliness Scale

The UCLA Loneliness Scale (Russell et al., 1980) is a 20-item self-report measure of trait loneliness. Participants are asked to indicate how often each of the statements listed is descriptive of themselves, ranging from 1 = *never* to 4 = *often*. Higher scores are indicative of greater trait loneliness. Internal consistency in the current study was excellent (*α* = 0.95).

#### State Loneliness

To measure state loneliness a single item visual analog scale was used. The scale consisted of a horizontal line with a slider bar, and endpoints labeled as “*not at all*” and “*very much.*” Participants were asked to slide the bar to the point on the line that best represents how they felt in that moment in regards to the following statement: “I feel lonely.” Responses to the scale could range from 0 to 100, with higher scores indicating greater loneliness.

### Procedure

Eligible participants could view and sign up for the study online through an online experiment management system. Upon sign up, participants gave their informed consent online. Participants then completed demographic questions and self-report measures of state and trait feelings of loneliness, general mattering, mattering to others, anti-mattering, fear of not mattering, as well as several other questionnaires not pertinent to the current study. Participants completed the measure of state loneliness prior to all other questionnaires, so that state loneliness scores were not influenced or primed by questions on the trait loneliness or mattering measures. Participants then received online debriefing and partial course credit for their participation. This study’s research protocol was approved by a university’s ethics review board and conforms to the standards of the Canadian Tri-Council research ethics guidelines.

### Data Analysis

All statistical analyses were conducted using SPSS version 25. Two separate multiple regression analyses were conducted to determine how general mattering, mattering to friends, anti-mattering, and fear of not mattering were related to both state and trait loneliness. A *priori* power analysis was conducted to determine the sample size needed to provide sufficient power (0.80) to detect a moderate effect size (0.25) at a significance level of alpha 0.05 for a multiple regression *F*-test analysis, using G*Power 9 (Faul et al., 2007). The power analysis indicated that the minimum sample size needed would be 53 participants, which we exceeded.

## Results

### Bivariate Correlations

Table 1 presents the means and standard deviations, as well as the correlations among all study variables. The means obtained in terms of levels of general mattering and anti-mattering are comparable to those summarized elsewhere (see Flett, 2018). It can be seen that state and trait loneliness are correlated substantially but not to the extent that they are redundant. As for the measures assessing the mattering construct, the correlations in Table 1 indicates that there is a substantial emphasis on the importance of fear of not mattering. Specifically, the fear of not mattering with others showed significant negative associations with both general mattering (*r* = −0.44) and mattering to friends (*r* = −0.46) and it was associated positively to a comparable degree with anti-mattering (*r* = 0.44).

**Table 1:** Intercorrelations between state and trait loneliness, anti-mattering, and mattering-related variables.

Variable	1	2	3	4	5	6	*M*	*SD*
1. State loneliness	–						33.40	32.72
2. Trait loneliness	0.63^**^	–					21.42	14.42
3. Mattering to friends	−0.38^**^	−0.70^**^	–				38.32	11.25
4. General mattering	−0.37^**^	−0.50^**^	0.61^**^	–			14.21	2.95
5. Anti-mattering	0.26^**^	0.42^**^	−0.22^**^	−0.14	–		12.99	2.86
6. Fear of not mattering	0.37^**^	0.60^**^	−0.46^**^	−0.44^**^	0.44^**^	–	6.40	3.84

*N* = 172. Range of possible scores for each construct are as follows: state loneliness: 0–100; trait loneliness: 0–63; mattering to friends; 11–55; general mattering: 5–20; Anti-mattering: 5–20; Fear of not mattering: 0–15.

* *p* < 0.05;

** *p* < 0.01.

Regarding the associations between the indices of mattering and loneliness, it can be seen in Table 1 that state loneliness was negatively related to both general mattering and mattering to friends, with correlations of moderate effect sizes. Trait loneliness was also negatively related to both general mattering and mattering to friends, with correlations of large effect sizes, especially for mattering to friends. Both state and trait loneliness were positively related to anti-mattering and fear of not mattering, with stronger correlations for trait than state loneliness.

### Regression Analyses

Table 2 presents the results of a multiple regression in which general mattering, mattering to friends, anti-mattering, and fear of not mattering are regressed onto state loneliness and trait loneliness in separate analyses. The analysis predicting state loneliness was significant, *F* (4, 167) = 11.66, *p* < 0.001, and accounted for 22% of the variance. As seen in Table 2, and as expected, both general mattering and mattering to friends were significant negative predictors of state loneliness. However, anti-mattering and fear of not mattering were not significant predictors of state loneliness.

**Table 2:** Multiple regression analyses predicting state and trait loneliness.

	B	*SE*	*β*	*t*	*p*	95% CI
State loneliness						
General mattering	−1.98	0.99	−0.18	−2.01	0.04	[−3.93, −0.04]
Mattering to friends	−0.53	0.26	−0.18	−2.01	0.04	[−1.04, −0.01]
Anti-mattering	1.59	0.87	0.14	1.82	0.07	[−0.14, 3.32]
Fear of not mattering	1.14	0.74	0.13	1.54	0.12	[−0.32, 2.59]
Trait loneliness						
General mattering	−0.26	0.30	−0.05	−0.86	0.39	[−0.86, 0.34]
Mattering to friends	−0.64	0.08	−0.50	−8.02	0.00	[−0.80, −0.49]
Anti-mattering	0.93	0.27	0.19	3.46	0.00	[0.40, 1.46]
Fear of not mattering	1.00	0.23	0.27	4.43	0.00	[0.56, 1.45]

The same analysis was conducted to predict levels of trait loneliness. The analysis predicting trait loneliness was significant, *F* (4, 167) = 67.44, *p* < 0.001, and accounted for 62% of the variance. It is important to underscore here that in this sample, the mattering variables were substantially more relevant to trait loneliness than state loneliness. As seen in Table 2, mattering to friends was a significant and negative predictor of trait loneliness, but contrary to predictions feelings of general mattering was not a significant predictor. Further, as predicted, both anti-mattering and fear of not mattering were significant and positive predictors of trait loneliness. Mattering to friends proved to be the most robust predictor of trait loneliness.

## Discussion

The purpose of the current study was to extend what is known thus far about the link between feelings of not mattering and loneliness. Our investigation focused on young women aged 18–25 years old, since recent research suggests they are the loneliest demographic group. Specifically, our aim was to examine how feelings of general mattering, mattering to peers, anti-mattering, and fear of not mattering related to young women’s self-reported state and trait loneliness. This was the first study to examine how mattering, anti-mattering, and fear of not mattering was related to both state and trait loneliness. This focus on multiple elements of mattering and both trait and state loneliness reflected our goal of conducting the most extensive study thus far on mattering and loneliness.

Overall, the pattern of results indicated that variables tapping mattering were related broadly to loneliness, as was expected, though they were much more relevant to understanding trait loneliness. To some extent, this could reflect in part the methodological constraints of very different self-report formats used to assess trait loneliness vs. state loneliness in the current study. Also, as we discuss in more detail below, there was also evidence attesting to the merits of examining various components of the mattering construct in terms of how they relate to loneliness.

Several key findings emerged in terms of how mattering is related to state and trait loneliness. First, as predicted, we found that low feelings of general mattering and mattering to peers were associated with greater feelings of state loneliness among young women. However, in regard to trait loneliness, only low mattering to peers, and not general mattering, was a significant predictor of greater trait loneliness. Our findings from the regression analysis qualified and extended the finding of Flett et al. (2016a) who found that low feelings of general mattering were associated with greater trait loneliness among young women. While both general mattering and mattering to peers was a significant predictor of state loneliness, we found that only mattering to peers was a significant predictor of trait loneliness among young women. These results highlight the importance of feeling valued and important to peers, on protecting young women from feelings of loneliness. This finding is in line with past research, which has found that social support and positive relationships with peers is a protective factor against feelings of loneliness among young women (Lee and Goldstein, 2016; Yildiz, 2016; Chang et al., 2017), and that greater perceived mattering to peers is associated with better well-being (Matera et al., 2020). Feelings of mattering to peers may be especially important to women who are emerging adults, as this is a time in their life when many young women are moving away from home to live independently to pursue work or start post-secondary education. Therefore, they may be losing immediate contact with family, and relying on support from friends during this time (Lee and Goldstein, 2016).

The second key finding was that greater levels of anti-mattering were associated with greater trait loneliness (but not state loneliness). Therefore, unsurprisingly, results indicated that feeling as if one does not matter at all to anyone, and feeling insignificant and invisible to others, is associated with greater trait feelings of loneliness. As such, these findings extend past complementary research, which has found that low social support and connection are associated with greater loneliness among young adults (Holt-Lunstad et al., 2010; Lee and Goldstein, 2016). The current findings add to this body of research, by noting that it is not just low social connection that is linked with increased feelings of loneliness, but also perceived subjective feelings of being insignificant to those with whom one does have social contact.

Finally, greater fear of not mattering was associated with greater trait loneliness (but not state loneliness). Fears over not mattering may reflect a ruminative cognitive style about the negative consequences of not mattering to others, or coming to matter less than one currently does. Chronic engagement in this kind of rumination may result in increased feelings of trait loneliness over time, as one consistently reflects on how awful it would feel to not matter to others. In turn, it is possible that those who are already lonely are predisposed to ruminating about these kinds of concerns. This finding is consistent with past research that has found that increased rumination is associated with greater loneliness (Gan et al., 2015; Borawski, 2019).

Taken together, the findings provide evidence for theoretical formulations of loneliness. Loneliness is not merely about being physically isolated, but rather the condition of *feeling* alone – feeling disconnected from others, and feeling that no one cares (Rokach, 2019), as well as feeling unimportant. In addition, the results further our clinical understandings of loneliness in the high risk group of young women by pointing to a need to focus on their concerns about whether they matter to their peers as well as their fears of not mattering. The problem of loneliness has been previously identified as a public health concern (Leigh-Hunt et al., 2017) but one of the ironies of the current public health pandemic crisis is that it has now heightened awareness to mobilize community efforts to reach out in novel ways to our most isolated and loneliest individuals (Flett and Zangeneh, 2020). With respect to the heightened risk of young women, this may involve not only “connecting” using social media, but finding ways to deepen these connections, not just through the sheer number of “friends” accumulated on social media (Hood et al., 2018) but through meaningful listening and discovering ways of reaching out that show kindness, compassion and true caring for others.

There are several implications that follow from the current evidence of the extensive and robust links between loneliness and feelings and fears of not mattering. At a theoretical level, conceptual models of phenomena such as the link between loneliness and physical health problems should consider how loneliness and feelings of being unimportant may combine to produce outcomes from a mediator or moderator perspective. Similarly, at a practical level, interventions designed to enhance the mental health and degree of interpersonal relatedness of profoundly lonely people should perhaps consider whether loneliness is accompanied by a sense of having little perceived value to other people. Both themes need to be addressed in clinical and counseling interventions. Of course, longitudinal or experimental research on the relationship between mattering and loneliness would better help to inform these interventions, as the current data only examined cross-sectionally how mattering is related to loneliness and cannot discuss whether this relationship is causal or not.

Like all research, the findings of the current study must be taken into consideration with some limitations. First, the data collected were based on self-report responses, which are always prone to subjective biases. Second, it was a limitation that the mattering to friends and anti-mattering measures had somewhat low reliabilities in the current study, which could have reduced correlations and the relation between anti-mattering on state loneliness. Additionally, the study design was cross-sectional and therefore the directionality of the relationships examined cannot be determined. While we proposed that low mattering, high anti-mattering, and fear of not mattering to others are correlates of loneliness that could increase risk for loneliness, it is also possible that loneliness could be a risk factor for decreased feelings of mattering, as well as increased anti-mattering and fear of not mattering. Longitudinal research is therefore needed to establish the directionality of the relationships examined, or experimental research to determine causality. While it was a strength of the study that we measured both state and trait loneliness and differentiated between two types of mattering, we only measured loneliness and mattering at one timepoint. Indeed, state loneliness was measured at a time prior to mandated physical isolation due to a public health crisis and most likely the ratings would have been quite different compared to a period of physical quarantine. Longitudinal research should be conducted that examines if mattering can predict loneliness over time, or vice versa. Also, it was the strength of the study that we investigated correlates for loneliness among young women, which is an under-researched demographic group in the loneliness literature. However, while our study only examined relations between loneliness and mattering in young women, we are confident that the findings of the current study would replicate among other demographic groups such as adolescents or the elderly, who also experience significant feelings of loneliness (Rokach, 2000; Fazio, 2009). In particular, feelings of mattering and fears of not mattering may be an especially important construct to study among the elderly, who often lose important social contacts as they age and experience greater loneliness. Past research has documented that loneliness increases from middle adulthood to old age, and that the loss of social contact through widowhood, having no spouse or cohabiting partner, and little contact with friends was associated with greater loneliness among the elderly (von Soest et al., 2020). Further, qualitative research among the elderly has documented that many elderly persons report gloomy feelings about not mattering and aching loneliness, which attributed to no longer having children who were dependent upon them and to losing their occupations through retirement (van Wijngaarden et al., 2015). Therefore, fears of not mattering may also be an especially relevant construct to study among those who are approaching old age and facing retirement. Feelings of mattering and fears of not mattering would also be relevant constructs to examine among adolescents who also report increased feelings of loneliness from childhood (Laursen and Hartl, 2013). Adolescence is a time where teens are trying to establish an identity separate from parents and where peer relations become increasingly important to self-development. Therefore, mattering to peers may be especially important in protecting against feelings of loneliness in adolescence (Laursen and Hartl, 2013).

In summary, the results of the current study confirmed that a reduced sense of mattering, especially to peers, and an increased sense of anti-mattering or fear of not mattering to others is associated with greater trait loneliness. Additional results showed that a reduced sense of general mattering and mattering to peers was associated with greater state loneliness. Overall, our results suggest that having a reduced sense of being significant and cared for by others could lead to state and trait feelings of loneliness among young women. These findings highlight the importance of young women forming strong social relationships, particularly with peers who make them feel important and cared for, in order to protect themselves against feelings of loneliness. The current findings suggest that bolstering feelings of mattering through therapy and other treatment avenues may help young women who are seeking to overcome feelings of state and trait loneliness.

## Data Availability Statement

The datasets presented in this article are not readily available because the requesting source must be affiliated with an academic institution. Requests to access the datasets should be directed to the corresponding author jgoldber@yorku.ca.

## Ethics Statement

The current study was reviewed and approved by the Human Participants Review Committee at York University. The patients/participants provided their written informed consent to participate in this study.

## Author Contributions

SM collected the data and wrote the first draft under the direct supervision of JG in fulfillment of her graduate student academic breadth requirements with further supervisory committee membership guidance by GF. JG and GF supervised the data analysis and edited drafts of the manuscript. AR provided further editorial and literature review support. All authors contributed to the article and approved the submitted version.

### Conflict of Interest

The authors declare that the research was conducted in the absence of any commercial or financial relationships that could be construed as a potential conflict of interest.
